# Roles of contour and surface processing in microgenesis of object perception and visual consciousness

**DOI:** 10.2478/v10053-008-0088-y

**Published:** 2011-12-01

**Authors:** Bruno G. Breitmeyer, Evelina Tapia

**Affiliations:** 1 Department of Psychology, University of Houston, Houston, TX, USA; 2 Center for Neuro-Engineering and Cognitive Science, University of Houston, Houston, TX, USA

**Keywords:** conscious visual processing, contour, nonconscious visual processing, surface color, surface contrast, temporal dynamics

## Abstract

Developments in visual neuroscience and neural-network modeling indicate the existence of separate pathways for the processing of form and surface attributes of a visual object. In line with prior theoretical proposals, it is assumed that the processing of form can be explicit or conscious only as or after the surface property such as color is filled in. In conjunction with extant psychophysical findings, these developments point to interesting distinctions between nonconscious and conscious processing of these attributes, specifically in relation to distinguishable temporal dynamics. At nonconscious levels form processing proceeds faster than surface processing, whereas in contrast, at conscious levels form processing proceeds slower than surface processing. I mplications of separate form and surface processing for current and future psychophysical and neuroscientific research, particularly that relating cortical oscillations to conjunctions of surface and form features, and for cognitive science and philosophy of mind and consciousness are discussed.

## Introduction

*Metacontrast* is a type of backward visual masking in which the vi-sibility of a brief target stimulus is suppressed by a following brief mask stimulus that spatially is adjacent to or surrounds the target. The time interval between the onsets of the two stimuli is termed *the stimulus onset asynchrony* (SOA). In metacontrast, the suppression of target visibility depends critically on the target-mask SOA: Suppression is weak at very small SOAs; for instance, 0 ms, and at SOAs in excess of 150 ms, and strongest when SOAs range between about 20 and 80 ms. As will become evident below the exact SOA value yielding optimal suppression of target visibility depends on the criterion content (Kahneman, 1968; see also Breitmeyer & Ömen, 2006, Chapter 2). We limit ourselves to several recent findings obtained in our laboratories and relate them (a) to findings – some quite recent – on the cortical architecture underlying visual perception, and (b) to issues concerning nonconscious and conscious visual information processing. Our recent findings, since they exploit metacontrast merely as a *method* to render stimuli more or less visible, do not constitute critical tests of extant theories of underlying metacontrast *mechanisms*, although any of the theories forseeably ought to accommodate them. Hence, although relevant to theories of visual masking, our approach allows us to reflect on the relation of these recent findings to current theories of visual consciousness. Here, instead of delving into specific mechanisms of metacontrast, we take it to be an effective experimental means or method for rendering attributes of stimuli invisible in order to probe the nature of nonconscious and conscious visual processing.

## Metacontrast and Criterion Content

Although there are so called *criterion-free methods* (e.g., the multiple-alternative forced-choice response method) for assessing psychophysical performance, in a typical perception experiment a human observer is instructed to respond to a stimulus according to some criterion. A stimulus presented to any sensory modality provides several sources of information. For example, when investigating the somatosensory system, a stimulus applied to the skin may have a certain size, texture, pressure, temperature, etc. Any of these attributes is a source of information that could be used to respond to the stimulus. When a particular source of information, by way of experimental instruction, becomes the basis of an observer’s responding to a stimulus, that source constitutes the observer’s criterion content. The psychophysical results obtained in a given study depend critically on the criterion content adopted. For example, during metacontrast one stimulus attribute of a visual stimulus such as its location or presence in the visual field may be accessible to conscious verbal report, while another such as its color or form may not (see Breitmeyer & Ömen, 2006, Chapter 8). However, an attribute that is inaccessible to conscious report may nonetheless register in the visual system and be accessible nonconsciously to a number of behavioral and motoric response systems (Dolan, 2002; Esteves & Öhman, 1993; Klotz & Neumann, 1999; Milner & Goodale, 1995, 2008; Weiskrantz, 1997).

**Figure 1.**
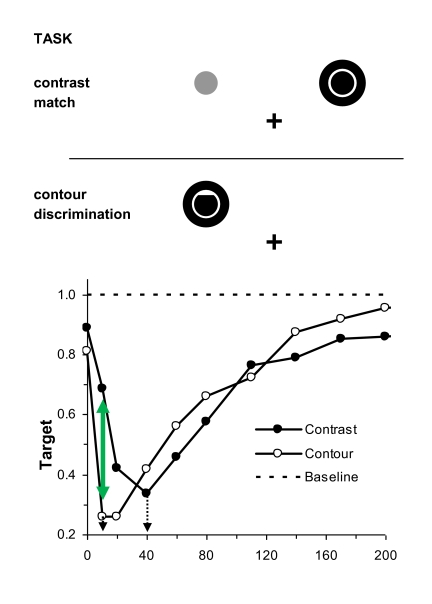
Top panel: The spatial layout of stimuli used to study a target’s surface and contour processing during metacontrast. In the contrast matching task, the target and comparison stimulus were presented slightly above and symmetrically to the right and left of fixation, and only the target was followed by a mask. The observer on any trial had to indicate which of the two stimuli, target or comparison, appeared darker. In the contour discrimination task, the target and following mask were both centred slightly above and either to the left or else to the right of fixation. The target could be a disk with either an upper contour deletion (as shown), a similar lower contour deletion, or no deletion. Here the stimulus display position and target shape was randomized over trials. Using a three-alternative forced-choice procedure, on any trial the observer had to indicate which of the targets was presented. Bottom panel: The normalized target visibility functions, relative to a baseline visibility of 1.0 obtained when the targets were presented without the following mask, shown separately for surface contrast and for contours, as a function of the stimulus onset asynchrony (SOA) between the target and the mask. Note (a) the difference of 30 ms between the optimal masking obtained for the contour discrimination and the contrast matching tasks (dotted arrows) and (b) the dissociation between contour and contrast visibilities at the SOSOA of 10 ms (dashed arrow). Adapted from “Meta- and Paracontrast Reveal Differences Between Contour and Brightness-Processing Mechanisms” by B. G. Breitmeyer, H. Kafaligönül, H. Öğmen, L . Mardon, S. Todd, and R. Ziegler, 2006, *Vision Research*, 46, pp. 2646, 2647.

 In a recent study, Breitmeyer et al. (2006) compared how metacontrast masking affects the perception of the luminance contrast (a surface feature) of a target to how it affects the perception of the target’s shape (a form feature). The methods and results of the study are illustrated in Figure 1. As shown in the upper panel of Figure 1, in one task, using a psychophysical tracking method, observers were asked to match the perceived luminance contrast of a black target’s surface relative to an unmasked comparison stimulus; in the second task the same observers were asked to identify one of three disk-like targets that differed in the shape delineated by their contours (a complete disk, a disk with an upper contour deletion as shown, and a disk with a lower contour deletion). Normalized visibilities of the targets for the two tasks are shown in the lower panel of Figure 1. Note that metacontrast, as expected, generally produces a decrease of the visibilities of the target’s surface contrast and of its form. What is moreover readily apparent is, first, that the SOA at which peak contour masking occurs is 10 ms, 30 ms shorter than the SOA of 40 ms at which peak surface contrast masking occurs. Consistent with similar latency differences of about 30 ms reported by Lamme, Rodriguez-Rodriguez, and Spekreijse (1999) and by Scholte, Jolij, Fahrenfort, and Lamme (2008) between cortical neural processing of the boundaries and the surface of a stimulus, our model simulations indicated that this SOA difference was due to a 30-ms faster processing of contour than of surface contrast. Second, as indicated by the solid green arrow in the lower panel of Figure 1, at the shortest SOAs ranging up to about 40 ms a dissociation existed between the contour and surface visibilities. All four observers who participated in the study, including the author, noted this dissociation. In particular, as indicated by the green arrow, at the SOA of 10 ms at which the form of the disk-like target was not seen or very difficult to see, the surface of the target appeared quite dark, matching about 70% of the contrast of the unmasked target. This indicates that at this SOA the mask strongly suppressed the processing of the target’s contour needed to discriminate the forms of the three disk-like targets while leaving largely intact the processing of its surface’s luminance contrast. This dissociation was reflected in the phenomenal appearance of a target area that was not completely filled out from its center to its contours, that is, of a dark target with distorted, missing or blurry contours.^1^

## Issues in the Study of Consciousness

 Although metacontrast as an experimental technique is easily definable, consciousness is not. Perhaps a first good attempt at an operational definition, provided by Searle (1992), is that *consciousness* is that state of an organism that is absent during, and is present after recovery from, deep anesthesia, or coma. As a point of departure, the following discussions assume that we are dealing with human observers who are in such a (conscious) state. Hence the focus will be on consciousness as a trait of cognitive contents (Stoerig, 2002). It is important to note that while many cognitive contents are conscious, many others are, or can be rendered, nonconscious. We take the conscious registration of stimulus information, that is, its cognitive contents, to constitute what has been referred to as *primary visual perception*, defined as “…our most basic subjective experiences of brightness and color that are sometimes referred to as ‘*qualia*’ [italics added]” (Pollen, 2008, p. 1991). *Conscious registration* thus refers to the subjective, phenomenal appearance of stimuli in the visual field. By that definition, terms such as *subliminal perception* or *unconscious perception* are misnomers. In their place we will use the term nonconscious vision or nonconscious visual processing. Although nonconscious vision or processing is most likely the only type of vision of organisms very low in the phylogenetic hierarchy, it can also arise in normal human observers exposed to one of several methods inducing transient “blindness” (Kim & Blake, 2005) and in a variety of neurological patients, such as those with blindsight (Persaud & Cowey, 2008; Weiskrantz, 1997).

In this article, we propose that visual consciousness is, in a more than metaphoric sense, superficial. To support this proposal, we follow up on the results of Breitmeyer et al.’s (2006) study revealing a distinction between two general kinds of subjective visual experiences accompanying the perception of objects. A visual object is characterized by boundaries or contours that delimit its geometric properties such as size, shape, and location in visual space and by surface properties such as color or luminance contrast, which fill the region within the contours. Perceived surface properties (such as color or lightness) are classic examples of purely *sensory qualia*; whereas the perceived form or shape attributes of visual objects are characterized by spatial extent and for that reason we henceforth refer to as *geometric qualia*.

The gist of the proposal is that the perception of geometric qualia, that is, the conscious registration of an object’s form attributes, such as orientation or curvature, depends necessarily on the conscious registration of sensory surface qualia, such as color. Without the superficial qualia there is no conscious visual apprehension of objects.

 Not all visual cognition of objects and their attributes depends on their being perceived. For example, in healthy observers one can experimentally induce transient stimulus blindness without affecting the processing of geometric attributes such as the shape, location or motion of a stimulus at a nonconscious level (Breitmeyer, Ömen, & Chen, 2004; Klotz & Wolff, 1995; Neumann & Klotz, 1994; Ömen, Breitmeyer, & Melvin, 2003; Wiesenfelder & Blake, 1991). Besides such instances of nonconscious vision in normal observers, several studies of blindsight in patients with damage to primary visual cortex have shown that when stimuli are presented to the affected field their location, motion, and wavelength can be discriminated without the accompanying registration of qualia (Cowey & Stoerig, 2001; Pöppel, Held, & Frost, 1973; Weiskrantz, 1997). Milner and Goodale (1995) also review a series of studies of cortically blind patients who nonetheless have access to geometric attributes of objects without conscious registration of corresponding geometric qualia. For instance, such a patient, while failing to report the conscious registration of objects, can appropriately adjust his or her grip aperture when reaching for objects of variable width. Hence, some visuo-cognitive functions, particularly those underlying the on-line control of skeletomotor actions (Milner & Goodale, 1995) can proceed “beneath the dashboard.” However, such control relies on short-lived cognitive contents that are continuously updated by information derived from the dynamically changing human-environment interactions and hence are not tied to perceptual information stored in (long-term or working) memory. In contrast, access to perceptual information is important in situations requiring the monitoring and resolving of conflicting interactive processes that accompany conscious guidance of skeletomotor action (Morsella, 2005). 

 The starting point of the ideas developed in the present paper, that the conscious registration of surface qualia, such as the color of visual stimuli, is necessary for the conscious registration of the stimuli as visual objects is not novel (e.g., Grossberg, 2003; Lamme et al., 1999; Ramachandran, 1992, 2003), and it appears so obvious as to warrant no further explanation. It has been intuited and expressed in one form or another by philosophers and cognitive scientists alike. For instance, regarding color, the philosopher of art John Hyman (2006), in his book, *The Objective Eye*, states that “…..there is *an intrinsic tie between color and sentience* [emphasis added], as there is between smell or taste and sentience, which *does not exist between sentience and shape* [emphasis added]” (p. 17). And shortly thereafter he elaborates that “…[one] cannot see the shape of a banana except by seeing its spatial boundaries, however fleeting and uncertain this experience may be. And [one] cannot see its spatial boundaries *except* [emphasis added] by seeing the differences of color that make it visibly distinct from its surroundings” (p. 18). Related views of the importance of surface features such as colors to our understanding of visual consciousness are expressed most explicitly by Stephen Grossberg’s (2003) claim that “surfaces are for seeing” (p. 19). Since standard definitions of *sentience* and *seeing* refer to conscious awareness, Hyman’s intuition and Grossberg’s model assert that our conscious awareness of shape depends on conscious awareness of surface properties such as color. Below, this thesis is elaborated within a neurocognitive framework that is consistent with existing psychophysical, neuroanatomical, and network modeling approaches to visual cognition. Implications of this thesis, some of which are not immediately obvious, for interpretations of extant findings and for further research on object perception also are discussed. 

## Neural Network Approaches to Object Vision

**Figure 2.**
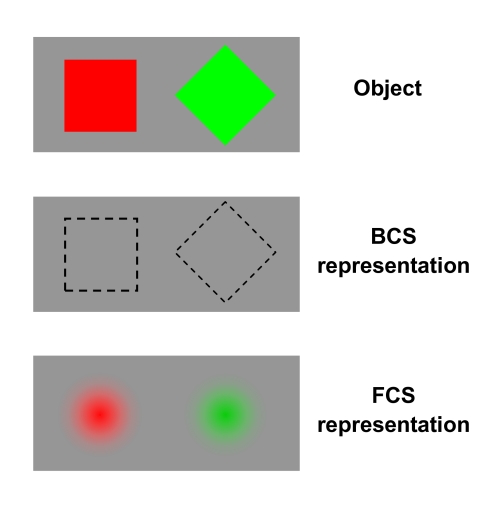
Depictions of two original stimulus objects (top panel), the implicit representation of their contours by the Boundary Contour System (BCS, middle panel), and their explicit representation via filling in of surface color/contrast by the Feature Contour System (FCS, bottom panel).

Biologically realistic models of vision (e.g., Biederman & Ju, 1988; Grossberg, 1994; Marr, 1982) incorporate separate processing modules responsible for determining the existence and location of boundaries, that is, the outer edges or contours, of objects and for filling in the surface properties within the boundaries of the object. The evolving versions of Grossberg’s model of visual processing (Grossberg, 1987, 1994; Grossberg & Yazdanbakhsh, 2005) provide particularly apt illustrations of these processes. The model incorporates what is known as the Boundary Contour System (BCS) and the Feature Contour System (FCS). The BCS specifies the existence and location of object boundaries. It delineates, for example, the two-dimensional form outline of an object. The FCS specifies the existence and type of surface features that fill in the area delimited by the BCS. When combined the perceived object is rendered in terms of its form attributes (the geometric qualia of orientation, width, length, curvature, etc.) and of its surface attributes (the sensory qualia of color, lightness or gray level, etc.). Thus we can perceptually distinguish two photo images of, say, an Anjou pear and a Bartlett pear by color; of others, such a green clover leaf and a green dandelion leaf by shape; and of still others, such as a banana and a pomegranate by color and shape. A schematic depiction of the contributions of the form-processing BCS and the surface-processing FCS to object perception is illustrated in Figure 2 for two objects, a square and a rhombus with differing, red and green, surface colors.

 An important aspect of the BCS is that its processing of form is implicit, that is, occurs at nonconscious levels (Grossberg, 2003), as indicated by the dashed lines delineating the contour outline of the objects. The interactive role of the BCS and the FCS in object perception has been stated as follows by Grossberg (1994) : 

A boundary that is completed within the segmentation system (denoted BCS) does not generate visible contrasts within the BCS. In this sense, *all boundaries are invisible*. Visibility is a property of the surface filling-in system (denoted FCS). *The completed BCS boundary can directly activate the object recognition system (ORS) whether or not it is visible within the FCS* [emphasis added]. In summary, a boundary may be completed within the BCS, and thereby improve pattern recognition by the ORS, without necessarily generating a visible brightness or color difference with in the FCS. (p. 59)

These model-based properties of the form-processing and the surface-processing systems resonate with a generally accepted notion that form or shape is processed before surface details are filled in (e.g., Humphreys, Cinel, Wolfe, Olson, & Klempen, 2000; Pessoa & De Weerd, 2003) and have important implications for our understanding not only of object recognition (Biederman & Ju, 1988; Marr & Nishihara, 1978; Ullman, 1984) but also of conscious and nonconscious visual processing. The gist of the present proposal is that the form-processing system, which extracts edge information, provides for the “deep” structure of visual consciousness while the surface-processing systems provides for its “surface” structure. The implications will be more fully explored after a description of the underlying neurobiological properties of vision that relate to the existence of and distinctions between the form- and surface-processing systems.

### Neurobiological substrate for form-processing and surface-processing systems in primate cortex

**Figure 3.**
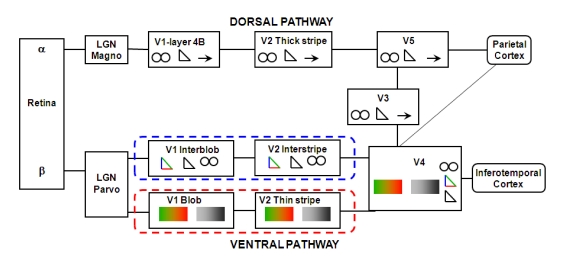
Schematic of the Boundary Contour System (BCS) and the Feature Contour System (FCS) in relation to two major, parvocellular (P) and magnocellular (M), visual pathways and their major projections, beginning respectively with retinal β and α cell, projecting via the lateral geniculate P and M layers to the primary visual cortex (V1). After V1 the M-pathway comprises the major, but not exclusive, dorsal projections to the parietal cortex; and similarly the P-pathway comprises the major, but not exclusive, ventral projections to the inferotemporal cortex. The dorsal projection is considered to comprise the “where” or the “vision-for-action” pathway; the ventral projection, the “what” or the “vision-for-perception” pathway. Note that in the ventral pathway, the FCS (outlined in red dashed lines) consists of the cortical P-pathway comprising the V1-blob / V2 (the secondary visual cortex)-thin stripe projections and beyond; the BCS (outlined in blue dashed lines) consists of the cortical P-pathway comprising the V1-interblob / V2-interstripe projections and beyond. The FCS processes only the wavelength and luminance properties (designated by the 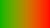 symbol and the 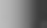 symbol) of an object’s surfaces. The BCS processes the object’s contours (designated by the symbol) defined either by isoluminant wavelength differences (designated by the 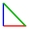 symbol) or by luminance differences (designated by the 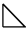 symbol). The dorsal pathway consists of the V1- layer 4B / V2-thick stripe projections and beyond. See text for further details. In all parts of the figure, the 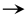 symbol designates ability to process motion direction, and the 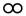 symbol designates ability to process binocular disparities. Adapted from “Concurrent Processing Streams in Monkey Visual Cortex” by E. A. DeYoe and D. C. Van Essen, 1988, *Trends in Neuroscience, 11, p. 223.*

Almost two and a half decades ago Livingstone and Hubel (1987, 1988) proposed separate cortical channels for the processing of form, color, movement, and depth of visual stimuli. According to this proposal, form and color are processed in the cortical parvocellular (P) pathways while depth and movement are processed by the cortical magnocellular (M) pathways. Along with the sharp distinction between M- and P-pathway, the strict subdivision of the cortical P-pathway into separate cortical P channels for color and for form, arising from the anatomically distinct blob and interblob areas in primary visual cortex (V1), respectively, is controversial (see e.g., DeYoe & Van Essen, 1988; Gegenfurtner, 2003; Kiper, 2003; Sincich & Horton, 2005b; cf. Figure 3). A significant number of orientation-selective form-processing neurons are also selective for wavelength (Friedman, Zhou, & von der Heydt, 2003; Gegenfurtner, 2003; Johnson, Hawken, & Shapley, 2001; Leventhal, Thompson, Liu, Zhou, & Ault, 1995; Thorell, De Valois, & Albrecht, 1984). Consequently there is no strict segregation of cortical form and color processing systems. As noted below, this lack of strict segregation turns out to be a useful property for the processing of visual stimuli. Nonetheless, accumulating evidence indicates that there are anatomically identifiable pathways and areas in the early and intermediate cortical object-processing systems that process primarily the surface properties of color and luminance on the one hand and the form properties of contour and edge orientation on the other (Conway, Moeller, & Tsao, 2007; Felleman, Xiao, & McClendon, 1997; Kinoshita & Komatsu, 2001; Lu & Roe, 2008; Roe & Ts’o, 1999; Sincich & Horton, 2005a; Wang, Xiao, & Felleman, 2007; Xiao, Casti, Xiao, & Kaplan, 2007; Xiao & Felleman, 2004; Xiao, Wang, & Felleman, 2003; Xiao, Zych, & Felleman, 1999). For instance, based on Felleman et al.’s (1997) work, cortical visual area 4 (V4), like visual areas 1 and 2 (V1 and V2), has separate neural compartments for shape and surface processing. Supporting this scheme, Girard, Lomber, and Bullier (2002) showed that reversible deactivation of V4 in macaque monkey can impair shape discrimination while leaving hue discrimination intact. 

 In line with proposals also suggested by others (Kiper, 2003; Roe & Ts’o, 1999), neurons tuned to color and orientation may be especially well suited for processing contours defined by wavelength differences. These would be especially important when the wavelength differences are at or near isoluminance. Thus, at isoluminance such neurons would contribute to the form-processing system. Non-oriented wavelength-specific neurons would instead contribute to the surface-processing system. Of course, luminance-defined borders and achromatic surface properties are also processed by the separate cortical form- and surface-processing systems. In a prior study of cortical chromatic processing, Xiao et al. (2003) showed that the thin stripes in V2, which receive input from V1’s color-selective blob areas, contain functional color-specific subregions in which variations of stimulus color are systematically mapped onto varying locations within the subregions. Recent findings reported by Xiao et al. (2007) indicate that cortical color maps exist as early as in the V1 blob areas. These could provide input to the spatially more extensive V2 thin-stripe color maps (Xiao et al., 2007). Wang et al. (2007) additionally found that adjacent to the color maps within the V2 thin stripes are neurons responding differentially to positive (light-on-dark) and negative (dark-on-light) luminance contrast. We propose, following Wang et al. (2007), that the thin stripes of V2 receive input from V1 blob areas and comprise neural modules for processing chromatic and achromatic surface properties of visual stimuli; whereas, neurons found in the adjacent V2 interstripes respond selectively to contour or edge orientation of stimuli and receive input from V1 inter-blob areas (see Figure 9 of Wang et al., 2007). 

### Evidence for separate but interactive form-processing and surface-processing systems in human vision

 Studies of neurological patients with specific impairments of visual perception reveal that the shape and surface properties of visual objects may be processed by dissociable cortical systems in humans as well as in primates. A number of studies (Barbur, Harlow, & Plant, 1994; Heywood, Cowey, & Newcombe, 1991; Kentridge, Heywood, & Cowey, 2004) have shown that despite loss of phenomenal hue perception of surfaces achromatopsic (cortically color-blind) patients can detect isoluminant chromatic edges or contours. Moreover, an achromatopsic patient investigated by Heywood, Wilson, and Cowey (1987), while grossly impaired in discriminating isoluminant hues, was able to discriminate different achromatic grays. This is consistent with Wang et al.’s (2007) findings in monkey indicating the existence of separate luminance and color processing within V2 thin-stripe surface-processing modules. In addition, recalling that selective loss of shape discrimination with intact hue discrimination was reported by Girard et al. (2002) when V4 was reversibly deactivated in macaque monkey, Zeki, Aglioti, McKeefry, and Berlucchi (1999) similarly showed that a patient, though all but form blind, was able to name objects’ colors. Moreover, even normal humans can perceive formless color, as shown by chromatic Ganzfeld stimulation (Cohen, 1958; Gur, 1989; Hochberg, Triebel, & Seaman, 1951). A chromatic Ganzfeld initially appears as a formless colored fog, which after several minutes of adaptation loses its phenomenal hue and appears as a neutral grey (Eigengrau). Like the results of neurological patients discussed above, in normal observers the original colored Ganzfeld percept and, after adaptation, its achromatic grey appearance are consistent with Felleman et al.’s (1997) and Wang et al.’s (2007) findings in monkey indicating the existence of separate color and luminance (grey-level) surface-processing modules. The combined studies of neurological patients and of normal observers in Ganzfeld stimulation thus indicate that in human vision, like in that of primates, form- and surface-processing systems can be separated from each other. 

 Grossberg’s LAMINART model (Cao & Grossberg, 2005; Grossberg & Swaminathan, 2004), extends the BCS and the FCS components of his FACADE model (Grossberg, 1994) by more fully elaborating their contribution to three-dimensional vision. The LAMINART model allows for descriptions not only of the form and surface properties of planar, two-dimensional objects but also of volumetric, three-dimensional objects with curved surfaces (two-dimensional manifolds). In order to construct a veridical object representation, the BCS and FCS must be able to communicate or interact appropriately with each other. Disturbances in these systems or in their interaction should therefore lead to distorted perceptions of object properties. Such perceptual distortions are demonstrated by some neurological patients whose contour-forming and surface filling-in interactive processes appear to be disturbed. In his review of deficits of color perception in neurological patients, Critchley (1965) reports cases in which the color of an object irradiates outward beyond its boundaries, sometimes at great distances from the boundaries of the object, and in which the boundaries of the object are often reported as fuzzy or blurred. In other cases the color of an object is perceived as not adhering to its surface, but instead as free-floating in space, in a plane distinct from that of the object and usually phenomenally located somewhere between the object and the patient. Related phenomena have been reported recently in a study of interocular continuous flash suppression (Tsuchiya & Koch, 2005) in normal observers conducted by Hong and Blake (2009). In this study rapidly changing achromatic (grey) Mondrian patterns were flashed to one eye while a stationary chromatic bar was presented to the other eye. Although observers failed to see the shape or form of the bar, they did report the color in a free-floating, cloudlike constellation. Both cases, the neurological symptoms and the experimental phenomena indicate that the form-processing BCS either does not construct or provide the necessary two- and three-dimensional spatial constraints for the filling-in process of the color-FCS system or that such constraints are not communicated to the FCS system. Consequently the object lacks proper color boundaries not only in the frontoparallel plane but also in depth. 

## The Spatiotemporal Dynamics of Form and Surface Processing I. Nonconscious Level

Although modified in a crucial way in the subsequent section, the discussion of the spatiotemporal dynamics of form and surface processing here takes as a starting point the generally agreed upon claim that the processing of form precedes the processing of surface features. As noted by Grossberg (1994, 2003) the processing of surface features (such as colors or luminance contrast) requires computations that compensate for variable intensities and wavelength compositions of the illuminant. Such *discounting of the illuminant* is necessary for yielding the two perceptual invariances realized in lightness and color constancy (Zeki, 1983a, 1983b; Zeki & Marini, 1998). Computations such as these may be more time consuming than those used to detect and bind contour features needed to establish an object’s form representation. Thus before surface features can render a stimulus visible by filling in its surfaces both its boundaries and its surface properties *must be processed at a nonconscious levels*.

It is known that without an intact primary visual cortex, V1, there are few if any qualia-rich contents of visual consciousness (Breitmeyer & Stoerig, 2006; Stoerig, 1996). Although V1 neural activity is necessary for conscious vision, there are cogent theoretical and empirical reasons for believing that it is not sufficient (Crick & Koch, 1995, 2003; Koch, 2004; Scheinberg & Logothetis, 1997). In particular, if, as Grossberg’s (2003) model posits, surfaces are for seeing and the FCS fills in the area bounded by the contours specified by the BCS only after the FCS has established lightness and color constancy, then the neural correlates of conscious vision must occur at or after the stage at which these constancy computations are completed. Although the existence of double-opponent (chromatically and spatially opponent) mechanisms in V1 provide the beginning stages of such computations (Gegenfurtner, 2003), the computations are not complete until at least the level of extrastriate area V4 (Heywood, Gadotti, & Cowey, 1992; Komatsu, Ideura, Kaji, & Yamane, 1992; Walsh, Carden, Butler, & Kulikowski, 1993; Zeki, 1983a, 1983b; Zeki et al., 1999). Hence, without further processing, neural activity in V1 cannot qualify as the sufficient neural basis of conscious object vision (Crick & Koch, 1995). Such activity can be defined as stimulus-dependent in so far as it can be elicited by the mere physical presence of a stimulus despite its not being perceived; whereas at higher levels in the ventral object-recognition pathway, percept-dependent neural activity tends to be elicited only when the stimulus is perceived (Scheinberg & Logothetis, 1997). In support of such a distinction between stimulus-dependent and percept-dependent activities, brain imaging studies of human observers indicate that conscious report of stimuli fails to occur without sufficient activation of higher levels of cortical processing (Beck, Rees, Frith, & Lavie, 2001; Dehaene et al., 2001; Lumer, Friston, & Rees, 1998). Activity at these higher levels may play a crucial role in conscious vision by reentering lower levels via top-down projections (Hochstein & Ahissar, 2002; Posner, 1994)

Given that contour and surface properties are processed at early, nonconscious levels such as V1, what is the evidence indicating that the processing of contour precedes the processing of surface properties at these levels? First, as noted electrophysiological recordings from V1 neurons in macaque (Lamme et al., 1999; Lee, Mumford, & Schiller, 1995) reveal separate processing of contour and surface properties of stimuli, with neural responses corresponding to surface properties lagging those corresponding to contour properties by about 30 ms (Lamme et al., 1999; see also Roelfsema, Lamme, Spekreijse, & Bosch, 2002). Related cortically evoked potential and psychophysical studies of human observers indicate slower surface than contour processing (Broder & Debruille, 2003; Caputo, Romani, Callieco, Gaspari, & Cosi, 1999; Romani, Caputo, Callieco, Schintone, & Cosi, 1999; Veser, O’Shea, Schröger, Trujillo-Barreto, & Roeber, 2008) and lower temporal resolution of surface than of contour processing (Rogers-Ramachandran & Ramachandran, 1998). Also consistent with slower processing of surface relative to contour properties are results of several psychophysical studies (Arrington, 1994; Breitmeyer et al., 2006; Elder & Zucker, 1998; Rossi & Paradiso, 2003).

 Here the recent recovery of target visibility from metacontrast masking reported by Ömen, Breitmeyer, Todd, and Mardon (2006) and the previously mentioned study by Breitmeyer et al. (2006) are additionally informative. One of the findings of the former study was that a primary mask(M1) can suppress the visibility of a target (T) even when M1’s visibility is itself suppressed by an aftercoming second mask (M2). Consequently, the neural mechanisms responsible for the metacontrast suppression (of T by M1) exert their effect at a nonconscious level of processing. The results of the latter study, which are illustrated in Figure 1, showed, as noted, that a metacontrast mask suppresses the contours of the target about 30 ms earlier than it suppresses surface contrast. Since the metacontrast suppression mechanism operates at a nonconscious level of processing (Ömen et al., 2006), it follows that *at this processing level*, the contours of the metacontrast-suppressed target were processed about 30 ms faster than its surface contrast. 

## The Spatiotemporal Dynamics of Form and Surface Processing II. Conscious Level

### A reversal of temporal order

The temporal priority of the processing of contour relative to surface at nonconscious cortical levels reverses as indicated by recent findings that abrupt changes of a stimulus’s color are perceived only about 40 ms earlier (Zeki & Bartels, 1998) or at best nearly simultaneously with (Viviani & Aymoz, 2001) the abrupt changes of its form. In a related study of interocular continuous flash suppression (Tsuchiya & Koch, 2005), Hong and Blake (2009) found that the color of a chromatic bar presented to the (temporarily) suppressed eye nearly always emerged into dominance (consciousness) before the orientation (form) of the bar was perceived. Rather than revealing a contradiction or inconsistency, we take these results to actually point out a key feature of the transition from nonconscious, pre-perceptual phase to a conscious, perceptual phase of form processing. According to the hypothesis articulated above that the conscious percept of form requires the conscious percept of a filled in surface, it follows that the form is *perceived only after* (*or as*) the filling in of its surface features such as color commences. For this reason the temporal dynamics of surface and contour processing, though characterized by a clear lag of surface processing at the nonconscious level, is characterized by a lead (or near synchrony) of surface processing at the conscious level.

### A proposed relation between surface processing and conscious processing in vision

 Recent empirical and theoretical work (Bullier, 2001; Hochstein & Ahissar, 2002; Lamme, 2006; Lamme & Roelfsema, 2000; Supčr, Spekreijse, & Lamme, 2001), supports the strong hypothesis that cortical feedforward and reentrant feedback activities provide a neural distinction between nonconscious and conscious processing, respectively. Similar proposals have recently been made in relation to backward masking by Breitmeyer (2007) and VanRullen (2007) and are consistent with Grossberg’s (1994, 2003) and Rees’s (2008) assertion that much of the cortical object recognition system can be activated at nonconscious levels of visual processing. What we make explicit here is that such processing occurs in the cortical feedforward sweep of activity. Indeed, Fahrenfort, Scholte, and Lamme (2007) and Boehler, Schoenfeld, Heinze, and Hopf (2008) recently showed that in humans the suppression of visibility of a target by an aftercoming mask correlates not with reduction of early feedforward activation in visual cortex but rather with reduction of the later reentrant activation. 

 Along with Lamme (2006) we propose that cortical reentrant activation most strongly correlates with conscious vision. But in light of the above discussions, we additionally emphasize that since surface completion is the sine qua non of the conscious registration of visual stimuli, the same cortical reentrant activation that correlates with conscious registration of visual stimuli must also correlate with cortical surface completion processes. Thus, the form features of a visual stimulus that are processed nonconsciously (Breitmeyer, 2007; VanRullen, 2007) become conscious geometric qualia of visual objects only as or after the surface features/qualia are filled in. 

## Implications for Research in Visual Cognition, Neuroscience, and Cognitive Science

Like other theoretical approaches (e.g., Grossberg, 1994, 2003; Rossi & Paradiso, 2003) the present approach posits (a) earlier processing of form than of surface properties, however only at nonconscious levels. Specifically like Grossberg’s (1994, 2003) approach, it posits (b) that the “surface structures” of visual consciousness, that is, its sensory qualia, constitute prerequisites for access to the “deep structures”, that is, the formal geometric qualia, of visual consciousness. From (b) it moreover follows (c) that a perception of surface properties precedes (or is nearly simultaneous with) the perception of form at conscious levels. These aspects of the approach, as noted above, are consistent with a number of neurophysiological and psychophysical results.

### Brain imaging studies

 The approach taken here claims that the conscious registration of visual geometric qualia depends on the conscious co-registration of sensory surface qualia. Without the latter there is no conscious vision of objects. This, as we noted above, has strong implications for theories of visual consciousness and for research strategies directed at finding the neural correlates of conscious vision. Specifically, in future brain recording and brain imaging research on neural correlates of conscious vision in humans, it would be particularly fruitful to examine activities in those areas of visual cortex that process surface features such as luminance contrast and color. Recent functional magnetic resonance imaging (fMRI) using strong magnetic fields promise surprisingly good spatial resolution of specific cortically evoked activities (Sun et al., 2007). With such high resolution magnets it may, for example, be possible to look for fMRI correlates of surface processing in human extrastriate areas that are homologues or analogues of cortical regions such as those reported by Conway et al. (2007) and by Felleman and colleagues (Felleman et al., 1997; Xiao & Felleman, 2004; Xiao et al., 1999, 2003; Wang et al., 2007). Moreover, it would also be informative to investigate how form-processing and surface-processing regions of cortex interact. If, as Bar and Biederman (1999) have proposed, visual awareness of object identity is associated with activity at or beyond the anterior region (area TE) of inferotemporal (IT) cortex, one would expect the surface filling-out processes also to be completed at or beyond that level. 

Additionally informative would be the study of how higher-level cortical activities associated with conscious report of visual stimuli, such as the dorsal prefrontal and parietal areas (Beck et al., 2001; Dehaene et al., 2001; Lumer et al., 1998), are functionally connected with the surface-processing and form-processing regions. A useful approach to investigating the roles of these higher-level processes would be to exploit the misbinding of orientation and color attributes known to occur during binocular rivalry in normal observers (Hong & Shevell, 2006; Shevell, St.Clair, & Hong, 2008). For example, if a vertical grating composed of alternate orange and grey bars is presented to one eye and a horizontal grating composed of alternate blue and grey bars is presented to the other eye, observers might report either eye-specific rivalry in which the former or the latter grating alternate their respective periods of perceptual dominance and suppression. Here, contour orientation and color are perceptually correctly bound (or not misbound). However, observers often also report seeing a (vertical or else a horizontal) grating composed of alternate orange and blue bars. Here color and contour orientations are perceptually misbound. Studies using electro-/magneto-encephalographic and fMRI brain imaging techniques may provide useful information as to the cortical connectivity patterns underlying such rivalry-induced misbindings of color and orientation information.

### Temporal order and micro-consciousnesses?

On the basis of asynchronies in the perceptual registration of stimulus attributes such as form, color and motion, Zeki (see e.g., Moutoussis & Zeki, 1997; Zeki, 2005; Zeki & Bartels, 1998) has proposed the existence of separate modular micro-consciousnesses, one for each stimulus attribute. In contrast to this proposal, the present approach instead argues that there cannot be a consciousness of form separate from that of surface properties such as color. This assertion does not exclude the possibility of the misbinding of chromatic or achromatic surface features and form features that were noted above (Friedman-Hill, Robertson, & Treisman, 1997; Hong & Shevell, 2006; Humphreys et al., 2000; Humphreys & Riddoch, 1994; Robertson, 2003; Shevell et al., 2008). Even when, say, a color and form are misbound and yield an illusory conjunction, the claim being made is that in these cases there will be no conscious percept of the form without a prior filling in of the wrong or inappropriate color information. On the other hand as also noted above, conscious spatially diffuse registration of color can exist separately from, that is, without, conscious registration of form (Hong & Blake, 2009).

### Feature integration and object recognition

Neurophysiologically plausible models of visual object recognition assume that the earliest cortical form-selective representation of a visual object is in terms of line or edge orientation (Biederman, 1987; Hummel & Biederman, 1992; Marr, 1982; Treisman, 1988; Ullman, 1996). Conjunctions of these or other feature primitives like curvature, size, color and contrast are assumed to occur at subsequent processing levels (Biederman, 1987; Hummel & Biederman, 1992; Treisman, 1988). These models are consistent with evidence showing that later stages of the ventral cortical processing stream are selective for patterns of input that consist of progressively more complex conjunctions of simple features (Desimone, Albright, Gross, & Bruce, 1984; Hubel, 1988; Pasupathy & Connor, 2002; Tanaka, Saito, Fukuda, & Moriya, 1991; Tsunoda, Yamane, Nishizaki, & Tanifuji, 2001).

**Figure 4.**
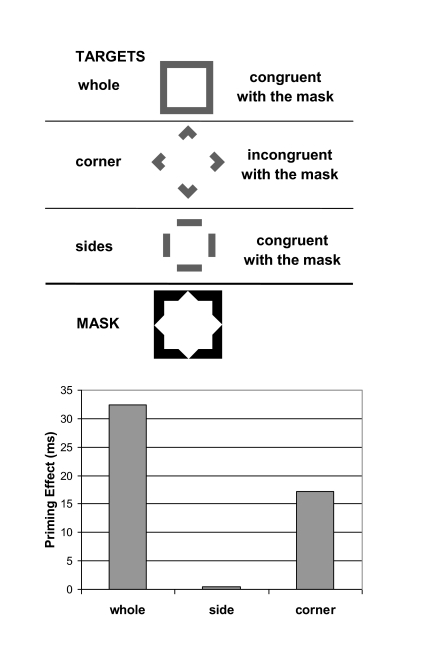
Upper panel: A schematic of several possible target stimuli followed, at an optimal masking SOA (the stimulus onset asynchrony) of 53 ms, by a surrounding metacontrast mask. The masked targets served as primes; the visible mask, as probe. The targets could either be presented in their entirety (whole), with only their vertices (corners), or only their side orientations (sides). Form features of the target could either be congruent or incongruent with those of the mask. The task of the observers was to respond as quickly and accurately as possible to the form of the mask, which could either be a square or a rhombus. Bottom panel: Priming effects for each of the three types of targets, obtained by subtracting the choice reaction time (RT) to the mask when the target and mask had congruent form features from the RTRT obtained when they had incongruent form features. Adapted from “Unconscious and Conscious Priming by Forms and Their Parts” by B. G. Breitmeyer, H. Öğmen, J. Ramon, and J. Chen, 2005, *Visual Cognition*, 12, pp. 722, 727.

 Above we noted that one can experimentally induce transient stimulus blindness without affecting the processing of geometric qualia such as the form, location, or motion of a stimulus at a nonconscious level (Breitmeyer, Ömen, Ramon, & Chen, 2005; Klotz & Wolff, 1995; Ömen et al., 2003; Neumann & Klotz, 1994; Wiesenfelder & Blake, 1991). Regarding form, Breitmeyer et al. (2005) used a masked priming paradigm in which a square- or a rhombus-shaped target served as the prime stimulus and an aftercoming and larger square- or rhombus-shaped metacontrast mask served as the probe stimulus. As shown in the upper panel of Figure 4, primes could be a whole square or rhombus or else consist of their parts, with the parts being either corners or else oriented sides. Probes were always a whole square or rhombus. Observers were required to respond as quickly and accurately as possible, by depressing one of two pre-designated keys, as to which probe was presented. Priming effects were defined as the difference between probe-reaction times obtained in the slower, incongruent (e.g., prime a square, probe a rhombus) and those obtained in the faster, congruent (e.g., prime a square, probe a square) conditions. The results of one experimental condition, shown in the lower panel of Figure 4, revealed that priming effects of the masked (invisible) target on choice reaction time to the (visible) probe/mask was strongest when whole targets served as primes, intermediate when the partial prime consisted of only its corners, and absent when the partial prime consisted of only its oriented line elements without corners. These results suggest that the nonconscious priming effect occurred at as late a level of processing at which conjunctions of simple form features, such as the conjunctions of oriented line elements comprising corners or conjunctions comprising holistic forms have been formed. This indicates that primitive form elements such as oriented lines or edges can be conjoined – such conjunctions resulting in, for example, corners and vertices, or indeed whole forms – *at a nonconscious level of processing*.

## Overview

The fundamental ideas discussed in this paper are that the visual processing of an object is partitioned along parallel channels into the processing of its location, its surface properties, and its form or contour properties. At cortical levels, an object’s location can be processed by the dorsal M-dominated pathway. Its form/contour properties are processed by the ventral P-dominated interblob/interstripe pathway, its surface properties are processed by the ventral P-dominated blob/thin-stripe pathway. At nonconscious levels the processing of form/contour properties precedes the processing of surface properties by several tens of milliseconds. However, if, as proposed, object vision ultimately depends on the filling in of surface properties, at conscious levels the asynchrony disappears or is reversed by several tens of milliseconds. Moreover, while form feature primitives can be conjoined at nonconscious levels establish representations of vertices, corners, etc. or the entire shape of an object, the conjunction of form and surfaces attributes appears to occur only at the conscious level of processing. These ideas furthermore explain a good portion of extant psychophysical findings regarding spatiotemporal aspects of object vision and suggest future directions of psychophysical and neuroscientific research on object vision.

## Footnotes

1 At longer SOAs, those above 40 ms, phenomenal reports of observers included a total or partial suppression of the target’s contours correlated with total or partial suppression of the surface contrast. However, despite not seeing the contours of the target, they were often able to infer the location of the contour features due to contour-feature transposition from the target to the mask (Herzog & Koch, 2001). Since apparent motion is the medium or carrier of feature transposition (Breitmeyer, Herzog, & Ömen, 2008) and since particularly these SOAs yield strong sensations of outward apparent motion from the target to the mask, one would expect to be able to infer features of the masked target contours (e.g, its contour cutoffs) by how they were transposed to the mask. This may explain why, at the SOAs of 40 ms and longer, the normalized visibility on target contour discrimination was slightly higher than that of target contrast.

## Author Note

Evelina Tapia is presently at Department of Psychology, University of Illinois at Urbana-Champaign, Champaign, IL, USA 61820.
